# Association of *MAPT* subhaplotypes with clinical and demographic features in Parkinson’s disease

**DOI:** 10.1002/acn3.51139

**Published:** 2020-08-07

**Authors:** Angela B. Deutschlander, Takuya Konno, Alexandra I. Soto‐Beasley, Ronald L. Walton, Jay A. van Gerpen, Ryan J. Uitti, Michael G. Heckman, Zbigniew K. Wszolek, Owen A. Ross

**Affiliations:** ^1^ Department of Neurology Mayo Clinic Jacksonville Florida; ^2^ Department of Neuroscience Mayo Clinic Jacksonville Florida; ^3^ Department of Neurology Brain Research Institute Niigata University Niigata Japan; ^4^ Division of Biomedical Statistics and Informatics Mayo Clinic Jacksonville Florida; ^5^ Department of Clinical Genomics Mayo Clinic Jacksonville Florida; ^6^ Neuroscience Track Mayo Graduate School Mayo Clinic Jacksonville Florida

## Abstract

**Objective:**

To determine whether distinct microtubule‐associated protein tau *MAPT* H1 subhaplotypes are associated with clinical and demographic features in Parkinson’s disease.

**Methods:**

A retrospective cohort study included 855 unrelated Caucasian patients with Parkinson’s disease who were seen by Movement Disorder specialists at the Mayo Clinic Florida between 1998 and 2016. The primary outcome measures were specific demographic and clinical features of Parkinson’s disease, including age at onset, disease progression, survival, motor signs, dementia, dystonia, dyskinesia, autonomic dysfunction, impulse control disorder, psychiatric features, REM sleep behavior disorder, restless legs syndrome, and Parkinson’s disease subtype. Specific clinical features were measured at the initial visit and most recent visit. These outcomes were assessed for association with *MAPT* H1 subhaplotypes, which were defined by six haplotype tagging variants.

**Results:**

Median onset age was 64 years (range: 22‐94 years); 548 (64%) of patients were male. Significant associations (*P* < 0.0029) were observed between *MAPT* H1b and orthostatic hypotension (OR = 1.72, *P* = 0.001); between H1j and rest tremor (OR = 0.15; *P* < 0.001) as well as REM sleep behavior disorder (OR = 3.87, *P* < 0.001); between H1r and bradykinesia (OR = 0.11; *P* < 0.001); and between H1v and restless legs syndrome (OR = 4.02, *P* = 0.002).

**Interpretation:**

Four *MAPT* H1 subhaplotypes, but not the H2 haplotype, were significantly associated with specific clinical features in Parkinson’s disease. *MAPT* haplotypic structure may explain some of the phenotypic variability in disease. Replication of our findings will be critical to fully resolve the Parkinson’s disease risk association signal at Chr17q21.

## Introduction

Parkinson’s disease (PD) is one of the most common movement disorders globally.^1^ PD is clinically characterized by four motor cardinal signs, namely, bradykinesia, rest tremor, rigidity, and postural instability. In addition, nonmotor features including autonomous signs (orthostatic hypotension, gastrointestinal and urogenital abnormalities), psychiatric features (impulse control disorder, ICD, depression, hallucinations), and cognitive decline (ranging from minimal cognitive impairment to dementia) have a significant impact on the quality of life in patients with PD. Due to the large phenotypic variability, independent and distinct subtypes of PD have been defined using different approaches.^2^, ^3^ We recently described four distinct subtypes in the large Mayo Clinic Florida cohort, namely, the tremor dominant (TD), the akinetic‐rigid (AR), the gait difficulty (GD), and the mixed subtypes.^4^

We now have a greater understanding of the genetic heterogeneity that influences PD susceptibility and etiology. The latest PD genome‐wide association study (GWAS) has reported more than 90 genetic risk variants.^5^ The susceptibility variants associated with an increased PD risk include variants around the *MAPT* gene, which encodes microtubule‐associated protein tau.^5^ The genomic architecture in the region spanning *MAPT* on chromosome 17q21 is highly complex with a ~1.8 Mb block of linkage disequilibrium (LD; a non‐recombining 900Kb inversion) and is defined by two common haplotypes, H1 and H2. Single nucleotide polymorphisms (SNPs) distinguish H1 and H2, and H1‐specifc SNPs generate a number of common *MAPT* H1 subhaplotypes.^6^, ^7^ The H1 subhaplotypes have been linked with an increased risk for several neurodegenerative diseases, including PD,^8^, ^9^, ^10^, ^11^ dementia with Lewy bodies,^12^ multiple system atrophy^12^ and progressive supranuclear palsy^6^, ^7^, ^13^; H2 haplotype decreases the risk for these neurodegenerative diseases.

In addition to studies estimating the increased risk of PD that is associated with *MAPT* H1, some studies have assessed associations between *MAPT* H1/H2 haplotypes and selected features, mainly cognitive function,^14^, ^15^, ^16^, ^17^, ^18^, ^19^ age at onset (AAO),^20^, ^21^, ^22^ PD subtype,^23^, ^24^ and progression.^25^ Therefore, the aim of this study was to assess the associations of specific *MAPT* subhaplotypes with detailed clinical features of PD in a large patient cohort collected and characterized at the Mayo Clinic Florida.

## Methods

### Study population

Patients with PD who were seen at the Mayo Clinic in Jacksonville, Florida, between July 1998, and December 2016, were included in this study. Diagnosis of PD was made according to standard criteria.^26^ All patients were Caucasian and unrelated to each other. Carriers of known pathogenic mutations for PD in *SNCA, LRRK2*, *VPS35*, *PARKIN*, *PINK1*, and *DJ1* were excluded. We did not make any exclusions based on presence of risk‐alleles for PD susceptibility variants (e.g., GWAS hits or *GBA* mutations). All patients provided blood samples for genetic testing after providing informed written content and this study was approved by the Mayo Clinic Institutional Review Board.

### Data collection

Demographic data and detailed clinical information were extracted from patients’ charts for each participant, as described previously (Table 1).^4^ Clinical data were collected during the initial visit (all patients) and the most recent visit (i.e., the last visit for patients who presented more than once, and the initial visit for patients with only one visit). Furthermore, patients were classified into one of the four following PD subtypes according to their initial evaluation: tremor dominant (TD), akinetic rigid (AR), gait difficulty (GD), and mixed.^4^

**Table 1:** Demographic and clinical data

Variable	Summary (N=855)
Age at PD onset (years)	64 (22, 94)
Sex (male)	548 (64.1%)
Initial visit	
Age (years)	69 (28, 97)
Disease duration (years)	3 (0, 38)
Medication includes levodopa	394 (46.1%)
Good levodopa responsivity	362 (91.9%)
Bradykinesia	818 (95.7%)
Rigidity	819 (95.8%)
Postural instability	181 (21.1%)
Resting tremor	590 (69.0%)
Postural tremor	199 (23.3%)
Kinetic tremor	61 (7.1%)
Dementia	4 (0.5%)
Dystonia	83 (9.7%)
Dyskinesia	87 (10.2%)
Autonomic dysfunction (GI, UG)	519 (60.7%)
Impulse control disorder	9 (1.1%)
(Pseudo‐)hallucinations	43 (5.0%)
Depression	261 (30.5%)
Orthostatic hypotension	61 (7.1%)
REM sleep behavior disorder (RBD)	91 (10.6%)
Restless legs syndrome (RLS)	42 (4.9%)
Most recent visit	
Age (years)	72 (28, 98)
Disease duration (years)	6 (0, 48)
Medication includes levodopa	681 (79.6%)
Good levodopa responsivity	656 (96.3%)
Bradykinesia	834 (97.5%)
Rigidity	828 (96.8%)
Postural instability	265 (31.0%)
Resting tremor	545 (63.7%)
Postural tremor	106 (12.4%)
Kinetic tremor	34 (4.0%)
Dementia	62 (7.3%)
Dystonia	116 (13.6%)
Dyskinesia	194 (22.7%)
Autonomic dysfunction (GI, UG)	626 (73.2%)
Orthostatic hypotension	118 (13.8%)
Impulse control disorder	16 (1.9%)
(Pseudo‐)hallucinations	107 (12.5%)
Depression	308 (36.0%)
REM sleep behavior disorder (RBD)	104 (12.2%)
Restless legs syndrome (RLS)	55 (6.4%)
Survival information	
Death	316 (37.7%)
Follow‐up length after PD onset (years)	8 (1, 48)
PD subtype	
TD	371 (43.4%)
AR	241 (28.2%)
GD	88 (10.3%)
Mixed	155 (18.1%)
Rapid progression	262 (41.4%)
Dementia within 5 year of onset	35 (6.3%)
Falling within 5 years of onset	231 (37.0%)
Become dependent on a caregiver within 5 years of onset	51 (9.0%)

TD, tremor dominant; AR, akinetic‐rigid; GD, Gait difficulty; GI, gastrointestinal; UG, urogenital.

The sample median (minimum, maximum) is given for continuous variables. Information was available regarding rapid progression for n = 633 patients; information on dementia within 5 years of PD onset was available for n = 557, on falling within 5 years of PD onset for n = 625, and on becoming dependent on another person within 5 years of PD onset for n = 566 patients. Information on rapid progression was unavailable for n = 222 patients due to the combination of absence of these three characteristics and insufficient follow‐up length. Survival information was available for n = 838 patients.

Finally, data on rapid progression were collected. Rapid progression was defined as the occurrence of either falling due to postural instability, or dementia, or becoming dependent on another person (i.e., requiring assistance for daily life activities such as maintaining hygiene, dressing, eating; or living in a nursing home) within five years from PD onset. If none of these three criteria had been met and the patient was not followed for five years after PD onset, information regarding rapid progression was considered to be unavailable.

### Genetic analysis

DNA was extracted from peripheral blood monocytes using standard protocols. Six *MAPT* haplotype‐tagging single nucleotide polymorphisms (SNPs; rs1467967, rs242557, rs3785883, rs2471738, rs8070723, and rs7521) were genotyped to assess the most common *MAPT* H1 subhaplotypes as well as the *MAPT* H2 haplotype as described previously.^12^ Genotyping was performed using TaqMan SNP genotyping assays on a QuantStudio 7 Flex Real‐Time PCR system (Applied Bio‐systems, Foster City, CA, USA; primer sequences available upon request). Genotype calls were made using TaqMan Genotyper Software v1.3 (Applied Bio‐systems, Foster City, CA, USA). Genotype call rates were 100% for each variant for all patients included. There were no departures from Hardy‐Weinberg equilibrium (all *P* > 0.01). Allele and genotype frequencies for each *MAPT* variant are provided for PD patients in Supplemental Table S1. Seventeen different *MAPT* haplotypes (16 H1 subhaplotypes and the H2 haplotype) were observed in ≥1% of individuals (Table 2).

**Table 2:** *MAPT* haplotypes observed in ≥1% of patients with Parkinson’s disease in association analyses

Haplotype (n = 17)	Haplotype frequency	*MAPT* variant					
		rs1467967	rs242557	rs3785883	rs2471738	rs8070723	rs7521
H1b	19.0%	G	G	G	C	A	A
H1c	12.9%	A	A	G	T	A	G
H1d	8.0%	A	A	G	C	A	A
H1e	8.6%	A	G	G	C	A	A
H1f	1.2%	G	G	A	C	A	A
H1g	2.0%	G	A	A	C	A	A
H1h	3.4%	A	G	A	C	A	A
H1i	4.5%	G	A	G	C	A	A
H1j	1.2%	A	G	G	C	A	G
H1l	3.1%	A	G	A	C	A	G
H1m	3.3%	G	A	G	C	A	G
H1o	1.6%	A	A	A	C	A	A
H1r	1.6%	A	G	G	T	A	G
H1u	2.6%	A	A	G	C	A	G
H1v	1.6%	G	G	A	T	A	G
H1y	2.1%	A	A	A	T	A	G
H2	16.7%	A	G	G	C	G	G

### Statistical analysis

Associations of six variant *MAPT* haplotypes with demographic and clinical features collected were evaluated using score tests of association,^27^ where haplotypes that occurred in less than 1% of patients with PD in the given association analysis were excluded. Specifically, score tests of association were performed under a linear regression framework (age at PD onset), under a logistic regression framework (dichotomous presence/absence features), and under a Cox proportional hazards regression framework (survival after PD onset). Clinical features were assessed for associations with *MAPT* haplotypes at both the first visit and the most recent visit. Tests were adjusted for sex when examining age at PD onset, for age at PD onset and sex when examining survival after PD onset and rapid progression, for age at the given visit, disease duration at the given visit, levodopa use at the given visit, and sex when assessing features that were examined both at the initial and most recent visit, and for age at initial visit and sex when examining PD subtypes. PD subtypes were categorized into four different dichotomous variables (i.e., TD vs other, AR vs other, GD vs other, and mixed vs other) for use in score tests of association. Regarding dichotomous clinical features, the given feature was not evaluated for associations with *MAPT* haplotypes if the rarer of these two categories occurred in fewer than 30 patients; these dichotomous clinical features were summarized descriptively only. This was done to avoid performing statistical tests with very low power to detect associations.

To adjust for multiple testing, we applied a Bonferroni correction separately for each clinical and demographic feature. Tests of association were performed for 17 different haplotypes, and therefore *P* ‐values < 0.0029 (i.e., 0.05/17) were considered as statistically significant. To limit the possibility of a type II error (i.e., a false‐negative finding), we also highlighted “suggestive” associations, which we considered to be those with a *P*‐value < 0.01. All statistical tests were two‐sided. Statistical analyses were performed using R Statistical Software (version 3.6.2; R Foundation for Statistical Computing, Vienna, Austria).

## Results

A total of 855 PD patients were included in this study. A subset of 613 patients had more than one visit. Patient characteristics are summarized in Table 1. Median age at PD onset was 64 years (Range: 22–94 years), male sex was most common (64.1%), and 41.4% of patients experienced rapid progression. TD was the most common PD subtype (43%), followed by AR (28%), mixed (18%), and GD (10%). After correcting for multiple testing, we did not observe any significant (*P* < 0.0029) or suggestive (*P* < 0.01) associations between *MAPT* H1 subhaplotypes and PD subtype; the strongest nonsignificant association was observed between the H1j subhaplotype and a lower risk of the TD subtype (OR = 0.16, 95% CI: 0.04‐0.76, *P* = 0.011). Of note, the H2 haplotype was not significantly associated with the TD subtype (OR = 0.91, *P* = 0.46), the AR subtype (OR = 1.00, *P* = 0.97), the GD subtype (OR = 0.73, *P* = 0.17), or the mixed subtype (OR = 1.37, *P* = 0.040).

Statistically significant and suggestive associations between *MAPT* H1 subhaplotypes and clinical features are displayed in Table 3. Significant associations were observed between the H1b haplotype and a higher likelihood of orthostatic hypotension at the most recent visit (OR = 1.72, 95% CI: 1.23‐2.40, *P* = 0.001), between H1j and a lower likelihood of rest tremor at the initial visit (OR = 0.15, 95% CI: 0.04‐0.48, *P* = 0.0003) as well as a higher likelihood of REM sleep behavior disorder (RBD) at the initial visit (OR = 3.87, 95% CI: 1.83‐8.20, *P* = 0.0004), between H1r and a lower likelihood of bradykinesia at the initial visit (OR = 0.11, 95% CI: 0.03‐0.34, P = 1 × 10^−7^), and between H1v and a higher likelihood of restless legs syndrome (RLS) at the initial visit (OR = 4.02, 95% CI: 1.15‐14.08, *P* = 0.002). Suggestive associations (*P* < 0.01) were noted for H1b (higher likelihood of dyskinesia at initial visit), H1f [higher likelihood of both dystonia and (pseudo)hallucinations at the most recent visit], and H1v (lower likelihood of depression at the initial visit). No significant or suggestive associations with H1 subhaplotypes were observed for age at PD onset, rigidity, postural instability, postural tremor, kinetic tremor, dementia, autonomic dysfunction, survival after PD onset, PD subtype, or rapid progression. The H2 haplotype was not significantly associated with any specific demographic or clinical feature.

**Table 3:** Associations between *MAPT* haplotypes and clinical features

Haplotype	Clinical feature haplotype is associated with	Haplotype frequency (%)		OR (95% CI)	*P*‐value
		Patients with the given clinical feature	Patients without the given clinical feature		
Significant associations (*P* < 0.0029)					
H1b (GGGCAA)	Orthostatic hypotension at most recent visit	26.8%	17.6%	1.72 (1.23, 2.40)	0.001
H1j (AGGCAG)	Rest tremor at initial visit	0.4%	2.9%	0.15 (0.04, 0.48)	0.0003
H1j (AGGCAG)	RBD at initial visit	3.3%	1.1%	3.87 (1.83, 8.20)	0.0004
H1r (AGGTAG)	Bradykinesia at initial visit	1.2%	8.4%	0.11 (0.03, 0.34)	1 × 10^‐7^
H1v (GGATAG)	RLS at initial visit	7.4%	1.4%	4.02 (1.15, 14.08)	0.002
Suggestive associations (*P* < 0.01)					
H1b (GGGCAA)	Dyskinesia at initial visit	26.9%	18.2%	1.84 (1.20, 2.82)	0.004
H1f (GGACAA)	Dystonia at most recent visit	3.7%	0.8%	4.96 (1.49, 16.54)	0.006
H1f (GGACAA)	(Pseudo)hallucinations at most recent visit	2.9%	1.0%	4.55 (1.22, 16.89)	0.009
H1v (GGATAG)	Depression at initial visit	0.0%	2.3%	N/A^1^	0.007

RBD, REM sleep behavior disorder; RLS, restless legs syndrome; OR, odds ratio; CI, Confidence interval.

P‐values result from score tests of association. Tests involving specific features at initial and most recent visit were adjusted for age at the given visit, disease duration at the given visit, levodopa use at the given visit, and sex. The order of the variants in defining the haplotype alleles is rs1467967, rs242557, rs3785883, rs2471738, rs8070723, and rs7521. ^1^Estimation of OR was not possible since estimated haplotype frequency in patients with depression at the initial visit was 0.0%.

## Discussion

The H1 haplotype on Chr17q21 containing *MAPT* is one of the most significant GWAS risk factors for PD and can be divided into approximately 20 different common subhaplotypes. Herein, we have identified five significant associations between H1 subhaplotypes and clinical features of PD, specifically H1b (higher risk of orthostatic hypotension), H1j (lower risk of rest tremor; higher risk of RBD), H1r (lower risk of bradykinesia), and H1v (higher risk of RLS). Interestingly, H1j has been associated with an increased risk of PD (compared to controls) in two previous studies,^9^, ^11^ and therefore the protective association with resting tremor in our study suggests this association is unrelated to tremor phenotype. Although not significant, the H1j haplotype has also been associated with an increased risk of RBD (OR = 2.16, *P* = 0.21),^11^ consistent with the findings of our study. Other than a nonsignificant trend toward an association between H1v and a lower risk of PSP (OR = 0.44, *P* = 0.06), the H1b, H1r, and H1v haplotypes have not been associated with other neurodegenerative disorders with a similar clinical or neuropathological phenotype to PD, such as PSP, MSA, or DLB.^12^, ^13^, ^28^ Though it will be important to validate the findings of our study, future studies examination associations of H1 subhaplotypes with clinical features of other neurodegenerative diseases will also be important.

We did not observe any associations between *MAPT* haplotypes and PD subtype. In a previous study of 46 tremor dominant (TD) and 135 non‐TD PD patients,^24^ the H1h subhaplotype occurred at a significantly higher frequency in the non‐TD PD patients compared to controls (7.4% vs2.6%) when compared with controls, whereas the difference between TD patients and controls was not statistically significant (5.3% vs2.6%). However, the lack of significance for the latter comparison may be due to limited power, and indeed the 7.4% and 5.3% frequencies for the TD and non‐TD patients are fairly similar, pointing toward a lack of association with PD subtype as found in our study. This previous study also observed a higher frequency of H2 carriers in the TD compared to non‐TD patients (26% vs13%), although no direct statistical comparison was made. In our study that included 371 TD patients, H2 was not associated with the TD PD subtype (OR = 0.91, *P* = 0.46), suggesting that this previous finding may be due to the much smaller number of TD patients and a correspondingly imprecise haplotype frequency. The H2 haplotype was also not associated with any other clinical features in our study, which is consistent with the findings of recent large GWAS’ where no associations between clinical features and *MAPT* variants were identified.^20^, ^29^

The primary strengths of our study are the relatively large cohort size and number of clinical features examined. However, several limitations should be acknowledged. Regarding the PD disease‐related data assessed in our study, this did not include objective scores such as UPDRS, Hoehn and Yahr, MoCA, or NMSS as they are not routinely collected on all patients. Another important limitation is the rare nature of many of the *MAPT* H1 subhaplotypes observed, which results in limited power to detect associations with clinical features. Of note, we opted to correct for multiple testing separately for each phenotypic feature rather than across all features to achieve a reasonable balance between possibilities of type I (false‐positive) and type II (false‐negative) errors. Thus, replication of our findings will be important, particularly for findings considered to be “suggestive” where a less stringent p‐value threshold was utilized (*P* < 0.01). Finally other genetic variants that affect phenotypic presentation such as *GBA* mutations and GWAS hits were not examined for epistatic interactions and further genome‐wide interaction approaches are critical.

In conclusion, we found *MAPT* H1 subhaplotypes H1b, H1j, H1r, and H1v were associated with an altered risk for specific clinical features in patients with PD, including orthostatic hypotension, tremor at rest, RBD, bradykinesia, and RLS. Some degree of caution is warranted when interpreting our results due to the aforementioned rare nature of many of the H1 subhaplotypes, and correspondingly replication of these findings is warranted. Regarding possible molecular pathways, the *MAPT* H1 haplotype has been shown to be associated with increased *MAPT* RNA expression in the brain as compared to the H2 haplotype.^30^, ^31^ *MAPT* haplotypes induce alternative splicing and RNA expression levels of the most common *MAPT* isoforms in the brain.^32^ Examining the influence of specific H1 subhaplotypes on gene expression is crucial and a better understanding of the genomic architecture at the Chr17q21 locus, may help us understand how genetic factors influence the phenotypic heterogeneity observed in PD.

## Author Contributions

*Substantial contributions to conception or design of the work*: all authors. *Acquisition, analysis, or interpretation of data*: all authors. *Drafting of the manuscript*: A. B. Deutschlander, M. G. Heckman, O.A. Ross.* Critical revision of the manuscript for important intellectual content*: all authors. *Statistical analysis*: M. G. Heckman, Obtained funding: A. B. Deutschlander, Z. K. Wszolek, O.A. Ross. *Administrative, technical, or material support*: all authors. *Supervision*: A. B. Deutschlander, Z. K. Wszolek, O.A. Ross. *Final approval of the version to be published*: all authors. *Agreement to be accountable for all aspects of the work in ensuring that questions related to the accuracy or integrity of any part of the work are appropriately investigated and resolved*: all authors.

## Conflicts of Interest

Authors report no conflicts of interest.

## Supporting information


**Supplemental Table S1.** Allele and genotype counts and frequencies. This table provides genotype counts and allele frequencies for each of the six single nucleotide polymorphisms that are used to generate the *MAPT* subhaplotypes for each of the individual patients.
