# Supplementary material for: Association of MAPT subhaplotypes with clinical and demographic features in Parkinson’s disease
Source: Ann Clin Transl Neurol. 2020 Aug 7;7(9):1557–63. doi: 10.1002/acn3.51139 (PMC7480915; doi:10.1002/acn3.51139)
Supplement: Supplementary file 1 — Supplemental Table S1. Allele and genotype counts and frequencies. This table provides genotype counts and allele frequencies for each of the six single nucleotide polymorphisms that are used to generate the MAPT subhaplotypes for each of the individual patients. [file ACN3-7-1557-s001.docx]

**Supplemental Table S1: Allele and genotype counts and frequencies**

| Variant | Minor allele count and frequency | Major allele count and frequency | Genotype 1 count and frequency | Genotype 2 count and frequency | Genotype 3 count and frequency |
| --- | --- | --- | --- | --- | --- |
| rs1467967 | G: 613 (35.8%) | A: 1097 (64.2%) | AA: 353 (41.3%) | AG: 391 (45.7%) | GG: 111 (13.0%) |
| rs242557 | A: 695 (40.6%) | G: 1015 (59.4%) | GG: 316 (37.0%) | GA: 383 (44.8%) | AA: 156 (18.2%) |
| rs3785883 | A: 305 (17.8%) | G: 1405 (82.2%) | GG: 580 (67.8%) | GA: 245 (28.7%) | AA: 30 (3.5%) |
| rs2471738 | T: 381 (22.3%) | C: 1329 (77.7%) | CC: 514 (60.1%) | CT: 301 (35.2%) | TT: 40 (4.7%) |
| rs8070723 | G: 294 (17.2%) | A: 1416 (82.8%) | AA: 592 (69.2%) | AG: 232 (27.1%) | GG: 31 (3.6%) |
| rs7521 | A: 849 (49.6%) | G: 861 (50.4%) | GG: 227 (26.5%) | GA: 407 (47.6%) | AA: 221 (25.8%) |
